# 

**Published:** 1904-10-22

**Authors:** 


### NOTICE TO CORRESPONDENTS.

All MSS., Letters, Books for Review, and other matters intended for the Editor should be addressed The Editor, "The Hospital," The Hospital Buildings, 28 & 29 Southampton Street, Strand, W.C.

The Editor cannot undertake to return rejected MS., even when accompanied by stamped directed envelope.

**Notice to Advertisers and Subscribers.**

All Advertisements, Orders for Copies of The Hospital, and Business Communications should be addressed to **THE MANAGER (Not to the Editor),** The Hospital, 28 & 29 Southampton Street, Strand, London, W.C.

The Cost of Subscription, post free, is as follows:—Per week, 3½d.; per quarter, 3s. 3d.; per half-year, 6s. 6d.; per annum, 13s. Foreign and Colonial:—Per annum, 19s. 6d., payable, in all cases, in advance.

**NOTICE.—Vol. XXXVI. of "The Hospital" (April to September 1904), in handsome cloth binding, is now on sale at the office of this Journal, price 10s. 6d. Cases for binding the Numbers contained in this Volume 2s. Index to Vol. XXXVI., 3½d., post free.**

**The Monthly part for October is now ready. Price 1s., by post, 1s. 3d.**

### BOOKS RECEIVED.

Authors' Association.

"With the Notts Militia in South Africa."

Cassell and Co.

"The Student's Handbook of Surgical Operations." By Sir Frederick Treves.

Royal Doulton Potteries.

"Pictures in Pottery."

Walter Scott Publishing Company, Ltd.

"Wild Flowers."

Young J. Pentland.

"Nervous Affections of the Heart." By Dr. G. A. Gibson.

Church Missionary Society.

"Preaching and Healing," 1904.

### Scraps and Gleanings.

Mr. J. Halsey Morton has been appointed Assistant Secretary to the Mount Vernon Hospital for Consumption and Diseases of the Chest, Hampstead and Northwood, Middlesex.

Messrs. Edward Cook and Co. have been awarded the Grand Prix at the St. Louis Exhibition for their exhibit of toilet, medicated, and household soaps.

### Medical Appointments.

**Robert Y. Aitken**, M.D., F.R.C.S., Honorary Sugeon to the Blackburn and East Lancashire Infirmary. **F. A. Beattie**, L.S.A., Medical Officer of Health, Hoylandswaine Urban District. **J. S. Colquhoun**, L.R.C.P., and S.Edin., L.F.P.S.Glas., District Surgeon at Underberg, Natal. **J. F. Corson**, M.B. Ch.B.Vict., Resident Assistant Medical Officer, Salford Union Infirmary. **F. W. Ellis**, Junior Resident Assistant Medical Superintendent, St. Pancras Infirmary. **Alfred W. Esler**, M.D., M.Ch. M.A.O.R.U.I., Honorary Surgeon, Williamstown Hospital, Victoria, Australia. **R. W. Fell**, M.B., C.M., District and Workhouse Medical Officer of the Buntingford Union. **Albert A. Gunn**, M.B., Ch.B.Edin., Honorary Assistant Physician to the Blackburn and East Lancashire Infirmary. **G. Hardwicke**, District Medical Officer of the Scarborough Union. **R. E. Legat**, M.B., C.M.Edin., Public Vaccinator for the Cromer District of the Erpingham Union. **J. W. Lewis**, L.R.C.P.andS.Edin., L.F.P.S. Glas., Certifying Factory Surgeon for the Brynamman District of the counties of Carmarthen and Glamorgan. **H. S. Lister**, M.B., Ch.B.Vict., Second Resident Assistant Medical Officer, Manchester Workhouse. **T. E. Manning**, M.B., District Medical Officer of the Mitford and Launditch Union. **John M. H. Martin**, M.D., F.R.C.S., J.P. Honorary Consulting Surgeon to the Blackburn and East Lancashire Infirmary. **J. R. Mason**, L.R.C.P.andS.Edin., L.F.P.S.Glas., District Medical Officer, Newcastle-on-Tyne Union. **George Mitchell**, M.B., Ch.B.Aberd., Junior Assistant to the Professor of Physiology, Aberdeen University. **J. Halsey Morton**, Assistant Secretary, Mount Vernon Hospital for Consumption and Diseases of the Chest, Hampstead and Northwood, Middlesex. **Miss M. H. Naylor**, M.B., B.S.Lond., Junior Assistant Medical Officer at the Camberwell Parish Infirmary. **Thomas Redahan**, L.R.C.P.andS.Irel., Certifying Factory Surgeon for the Mohill District, county Leitrim. **James E. H. Sawyer**, M.A., M.D.Oxon, M.R.C.P., Pathologist at the General Hospital, Birmingham. **P. T. H. Stedman**, M.B.Lond., M.R.C.S., Medical Officer of Health, Wing Rural District. **Roland A. Stevenson**, L.R.C.P.Lond., M.R.C.S.Eng., Medical Superintendent at the London Open-Air Sanatorium, Pinewood, Wokingham, Berks. **S. W. Thompson**, M.R.C.S., L.S.A., District Medical Officer of the Greenwich Union. **F. E. Tylecote**, M.B., Ch.B. Vict. Senior Resident Assistant Medical Officer, Manchester Workhouse. **J. Christopher Wadmore**, L.R.C.P. Lond., M.R.C.S.Eng., Assistant Resident Medical Officer at the London Open-Air Sanatorium, Pinewood, Wokingham, Berks. **Alexander Watt**, M.A., M.B., Ch.B., D.P.H.Abdn., First Assistant to the Inspector of Asylums for Cape Colony. **F. Wolverson**, M.B., C.M.Glas., Medical Officer and Public Vaccinator, Aldridge Division of Walsall Union.

**"The Hospital" Institutional Library.**

**"Hospitals and Asylums of the World." 4 Vols., with a Portfolio of Plans, complete £12 12s.**

**"Burdett's Hospitals and Charities, 1904." 5s. net.**

**"Burdett's Official Nursing Directory." 8s. net.**

**"Cottage Hospitals, General, Fever, and Convalescent." 10s. 6d.**

**"Uniform System of Accounts for Hospitals and Public Institutions." New Edition. 4s. net.**

**"Hospital Expenditure: The Commissariat." 2s. 6d.**

**"A Legal Handbook for the Use of Hospital Authorities." 1s. 6d.**

**"The Cottage Hospital Case Book or Register of Patients." 10s. and 12s. 6d.**

**All these are published by the Scientific Press, Ltd., and may be obtained through any bookseller, or direct from the publishers, 28 and 29 Southampton Street, Strand, London, W.C.**

### Medical Appointments.

AN EXAMINATION OF CANDIDATES for entry into the Medical Department of the Royal Navy will be held on 14th November next and following days at Examination Hall, Thames Embankment.

The forms to be filled up by Candidates will be supplied on application to the Medical Director-General.

Admiralty,
30th September, 1904. 18 Victoria Street, S.W.
(53)

INFIRMARY MALE ASSISTANT MEDICAL OFFICER.

The Guardians of the Poor of the Parish of St. Marylebone are desirous of receiving applications for the above appointment at their Infirmary, Rackham Street, Ladbroke Grove Road, Notting Hill, W.

Candidates must be unmarried, have had previous hospital experience, and must be duly registered and qualified by law to practise both medicine and surgery in England, and must not exceed 30 years of age.

Salary £150 per annum, with Furnished Apartments, Rations, and Washing. The appointment, salary, and emoluments will be subject to the approval of the Local Government Board and to the terms of the Poor Law Officers' Superannuation Act, 1896.

Applications, endorsed "Application for the Appointment of Infirmary Assistant Medical Officer," stating age and present and past appointments, with copies of testimonials of recent date, to be forwarded to me on or before Tuesday, November 1st, 1904.

Personal canvassing is strictly prohibited.

By order.

HENRY T. DUDMAN,

Clerk to the Board.

Guardians' Offices,

Northumberland Street, Marylebone Road, W.,

October 18th, 1904. (1008)

### Books for Nurses.

**THE NURSING PROFESSION:**
**How and Where to Train.**
Edited by Sir Henry Burdett, K.C.B.
2/- net. 2/4 post free.

**A HANDBOOK FOR NURSES.**
By Dr. J. K. Watson.
5/- net. 5/4 post free.

**THE NURSES' PRONOUNCING DICTIONARY OF MEDICAL TERMS AND NURSING TREATMENT.**
2/- post free.

**A COMPLETE HANDBOOK OF MIDWIFERY.**
By Dr. J. K. Watson.
6/- net. 6/4 post free.

**PRACTICAL GUIDE TO SURGICAL BANDAGING AND DRESSINGS.**
By W. Johnson Smith, F.R.C.S.
2/- post free.

**NURSING IN DISEASES OF THROAT, NOSE, AND EAR.**
By P. MACLEOD YEARSLEY, F.R.C.S.
2/6 post free.

**ELEMENTARY PHYSIOLOGY FOR NURSES.**
By C. F. Marshall, M.D., F.R.C.S.
2/- post free.

**MASSAGE: The Swedish System and Nauheim Movements.**
By Axel. Grafstrom, B.Sc., M.D.
2/6 post free.

**THE MODERN NURSING OF CONSUMPTION.**
By Dr. Jane Walker.
1/- net. 1/1 post free.

**NURSING.**
**(An American Text-Book.)**
By Isabel Hampton.
7/6 net. 7/10 post free.

**THE BURDETT SERIES OF NURSING TEXT-BOOKS.**
**All Subjects.**
1/- post free.

**NEW CASE-BOOK—PRIVATE NURSING NIGHT AND DAY.**
Large size, 3d. net. 4½d. post free.

**CASE-BOOKS**
**For all Branches of Nursing.**

**CHARTS**
**and every Requirement for Nursing Treatment.**

**The Scientific Press, Ltd.,**
28 & 29 Southampton St., Strand, London, W.C.

*Publishers and Booksellers of every kind of Nursing Text-Books, Case-Books and Charts.*

**SEND FOR NEW CATALOGUE.**

